# Alterations in cortical volume and complexity in Parkinson's disease with depression

**DOI:** 10.1111/cns.14582

**Published:** 2024-02-08

**Authors:** Jiaying Yuan, Yujing Liu, Haiyan Liao, Changlian Tan, Sainan Cai, Qin Shen, Qinru Liu, Min Wang, Yuqing Tang, Xu Li, Jun Liu, Yuheng Zi

**Affiliations:** ^1^ Department of Radiology, The Second Xiangya Hospital Central South University Changsha China; ^2^ Clinical Research Center For Medical Imaging in Hunan Province Changsha China; ^3^ Department of Radiology, The First Affiliated Hospital, Hengyang Medical School University of South China Hengyang China

**Keywords:** depression, Parkinson's disease, structural magnetic resonance imaging, surface‐based morphometry (SBM), voxel‐based morphometry (VBM)

## Abstract

**Aims:**

The aim of this study is to investigate differences in gray matter volume and cortical complexity between Parkinson's disease with depression (PDD) patients and Parkinson's disease without depression (PDND) patients.

**Methods:**

A total of 41 PDND patients, 36 PDD patients, and 38 healthy controls (HC) were recruited and analyzed by Voxel‐based morphometry (VBM) and surface‐based morphometry (SBM). Differences in gray matter volume and cortical complexity were compared using the one‐way analysis of variance (ANOVA) and correlated with the Hamilton Depression Scale‐17 (HAMD‐17) scores.

**Results:**

PDD patients exhibited significant cortical atrophy in various regions, including bilateral medial parietal–occipital–temporal lobes, right dorsolateral temporal lobes, bilateral parahippocampal gyrus, and bilateral hippocampus, compared to HC and PDND groups. A negative correlation between the GMV of left precuneus and HAMD‐17 scores in the PDD group tended to be significant (*r* = −0.318, *p* = 0.059). Decreased gyrification index was observed in the bilateral insular and dorsolateral temporal cortex. However, there were no significant differences found in fractal dimension and sulcal depth.

**Conclusion:**

Our research shows extensive cortical structural changes in the insular cortex, parietal–occipital–temporal lobes, and hippocampal regions in PDD. This provides a morphological perspective for understanding the pathophysiological mechanism underlying depression in Parkinson's disease.

## Graphical abstract

The first row of pictures shows extensive cortical volume loss in Parkinson's disease with depression patients, primarily concentrated in the parietal–occipital–temporal lobes, parahippocampal gyrus, and hippocampus. The second row of pictures shows decreased gyrification index in the insula.

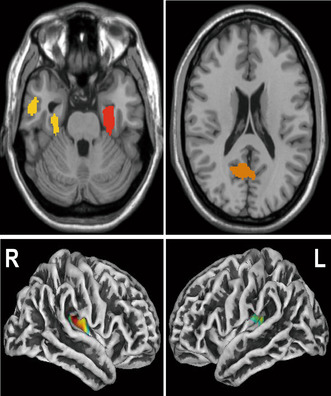

## 1 INTRODUCTION

Depression is one of the most common non‐motor and psychiatric symptoms experienced by individuals with Parkinson's disease (PD)^1^, ^2^ and it can occur at any stage of the disease course, affecting up to 38% of patients.^3^ Parkinson's disease with depression (PDD) not only causes negative emotions such as loss of interest, hopelessness, and lack of pleasure but also leads to cognitive and motor impairments, significantly impacting individuals' quality of life and increasing caregiving costs.^4^, ^5^ Therefore, investigating PDD is of substantial clinical significance. Advances in neuroimaging technology have provided numerous valuable non‐invasive scientific tools for studying the brains of individuals with PD‐related depression. Numerous studies have consistently identified both structural and functional brain abnormalities in PDD patients. Magnetic resonance imaging (MRI) studies of brain structure have shown reductions in gray matter volume (GMV) in regions such as the orbitofrontal cortex, temporal cortex, limbic system (comprising the amygdala, hippocampus, and cingulate gyrus), and cerebellum in PDD patients.^6^, ^7^, ^8^ Kostic et al.^9^ reported white matter volume (WMV) loss in the anterior cingulate gyrus and orbitofrontal gyrus of PDD patients, suggesting damage to nerve fibers in these areas. Cortical thickness studies have shown widespread reductions in the frontal, parietal, occipital, and temporal lobes in PDD patients.^10^, ^11^, ^12^ Functional brain imaging studies associated with PDD indicated increased spontaneous neural activity in the frontal lobe region and decreased functional connectivity within the prefrontal‐limbic system, contributing to the mechanisms of depression in PD.^13^, ^14^ Moreover, studies have reported abnormal synchronization of neuronal activity in multiple brain regions including the motor cortex and parieto‐occipital lobe, and extensive functional connectivity abnormalities at the whole‐brain level in PDD.^15^, ^16^ These findings collectively suggest widespread brain functional disturbances and structural alterations in PDD patients, with deficits in the prefrontal‐limbic system as a central feature.

Brain structural MRI studies, among the multimodality MRI available, are extensively utilized due to their intuitive, reliable, and stable findings. Structural MRI studies primarily include voxel‐based morphometry (VBM)^17^ and surface‐based morphometry (SBM),^18^ both of which objectively depict structural changes in the central nervous system, offering new perspectives for exploring the pathophysiological mechanisms, diagnosis, and prognosis evaluation of diseases. VBM uses 3D‐T1WI structural images to quantitatively analyze GMV and WMV on a voxel‐by‐voxel basis, effectively detecting local variations in brain morphology.^17^ Over recent years, VBM has gained prominence in investigating brain structure in neuropsychiatric diseases including PD and major depressive disorder (MDD).^6^, ^7^, ^8^, ^19^ SBM analysis is based on brain tissue segmentation and cortical reconstruction to calculate indices such as cortical thickness, gyrification index (GI), fractal dimension (FD), and sulcal depth.^18^ These indices quantify the morphological structure of the gray matter and estimate various characteristics of the cerebral cortex, with the latter three indices representing cortical complexity, referring to cortical folding characteristics. Notably, existing SBM studies on PD‐related depression have primarily concentrated on cortical thickness and have not extensively explored GI, FD, and sulcal depth. Studies of MDD have consistently concluded that cortical complexity‐related indices reflect early neurodevelopment and are a potential vulnerability marker for MDD, which is more sensitive than cortical thickness in reflecting cortical developmental abnormalities.^20^, ^21^ Early neurodevelopmental markers showed a significant correlation with clinical indicators of MDD (duration of illness and number of depressive episodes),^20^ and can also aid in predicting the efficacy of electroconvulsive therapy (ECT).^21^ Furthermore, research by Nixon et al.^22^ confirmed the link between functional connectivity and cortical GI, suggesting that abnormal folding in brain regions associated with emotional processing can lead to dysfunction in these regions, resulting in emotional processing disorders in patients.

The current body of research on gray matter structure in PD‐related depression is limited and shows inconsistent findings. The results of existing studies on GMV changes in PDD are mixed. An early study revealed reduced GMV in the left orbitofrontal gyrus, bilateral rectus gyrus, and right superior temporal region in PDD compared to Parkinson's disease without depression (PDND), and the study also found negative correlations between GMV reductions in these cortical and limbic regions (including the medial temporal cortex, anterior cingulate cortex, medial cingulate cortex, and parahippocampal cortex) and depression scale scores.^7^ Kostic et al.^9^ reported reduced WMV in the anterior cingulate gyrus and orbitofrontal regions but did not observe any alterations in GMV. In contrast, Mierlo et al.^6^ suggested a positive correlation between GMV reduction in the anterior cingulate gyrus and depression severity. The inconsistency may be attributed to several factors including variations in sample size, the severity of depression, and differences in the methods used to assess depressive symptoms. In addition, previous studies have focused on analyzing GMV or cortical thickness as single indices and have seldom explored changes in cortical complexity.

Our study aimed to investigate potential alterations in both cortical volume and complexity among PDD and PDND patients. To achieve a comprehensive exploration of structural brain changes, we employed a combination of VBM analysis and SBM analysis. This multifaceted approach provides a thorough exploration of the neuroimaging mechanisms that underlie depression in PD from a morphological perspective.

## 2 MATERIALS AND METHODS

### 2.1 Subjects and clinical assessment

This study received approval from the Medical Ethics Committee of the Second Xiangya Hospital of Central South University. All participants provided written informed consent. Primary PD patients who visited the Department of Neurology at our Hospital, from July 2019 to October 2022. were recruited, while healthy controls (HC) were recruited from the community.

All participants planned to take brain MRI, clinical data collection, as well as detailed neurological and psychiatric assessments. For PD patients, information such as age, gender, disease duration, age at onset, and years of education was recorded. The Unified Parkinson's Disease Rating Scale motor score (UPDRS III), Modified Hoehn‐Yahr stage (H‐Y), and Mini‐Mental State Examination (MMSE) were used to evaluate the motor status and cognitive function of PD patients. The Hamilton Depression Scale‐17 (HAMD‐17) was employed to assess the participants' depressive symptoms. Previous research has demonstrated that a cutoff score of 9/10 offers high sensitivity and specificity in detecting depression in PD patients.^23^ Based on the HAMD‐17 scores, PD patients were divided into two groups: the PDD group (HAMD‐17 score ≥ 10) and the PDND group (HAMD‐17 score < 10).

In total, 102 PD patients (54 PDND and 48 PDD) and 46 HC were initially recruited. After applying the exclusion and inclusion criteria, 77 PD patients (41 PDND and 36 PDD) and 38 HC were included in the final analysis.

The inclusion criteria for PD patients were as follows: (1) Meeting the 2015 Movement Disorder Society Clinical Diagnostic Criteria for Parkinson's disease (MDS‐PD Criteria),^24^ and being capable of undergoing MRI scanning and scale assessments; (2) right‐handedness; and (3) no use of anti‐PD medications within 12 h.

The exclusion criteria for PD patients were as follows: (1) Known specific pathological factors such as drug‐induced or infection‐related secondary parkinsonism or clinical diagnosis of the parkinsonism‐plus syndrome; (2) presence of significant structural brain lesions (such as severe head trauma, cerebrovascular disease, epilepsy, or history of neurosurgical procedures), or history of other neurologic or psychiatric diseases (such as schizophrenia or bipolar affective disorder). (3) Presence of cognitive impairment based on MMSE scores (MMSE score below 17 for illiterate individuals, below 20 for those with 1–6 years of education, and below 23 for those with seven or more years of education). (4) Use of antidepressant medications currently or before. (5) Patients' head movements were too large on magnetic resonance images (head movement larger than 2 mm) to affect data analysis.

The inclusion criteria for HC were as follows: (1) matched in terms of age, gender, and years of education with PD patients; and (2) right‐handedness.

The exclusion criteria for HC were as follows: (1) presence of significant structural brain lesions or other neurologic or psychiatric diseases; (2) presence of cognitive impairment based on MMSE scores; and (3) presence of depressive symptoms, indicated by a HAMD‐17 score ≥ 8.

### 2.2 MRI image acquisition

All participants underwent brain MRI scans using the same 3.0T MRI scanner (MAGNETOM Skyra; Siemens Healthineers) in the Radiology Department of our Hospital. High‐resolution T1‐weighted images were acquired using a head–neck combined coil. The scanning parameters were as follows: number of slices: 176; repetition time (TR): 1900 ms; echo time (TE): 2.01 ms; flip angle: 9°; field of view: 256 × 256 mm; voxel size = 1 × 1 × 1 mm; and slice thickness: 1.0 mm (no slice gap). The total scan duration was 8 min and 8 s.

### 2.3 Data processing

The data preprocessing involved the following steps: (1) Conversion of MRI images from DICOM format to NIFTI format using MRIcro software. (2) Utilization of the Cat12 toolbox implemented in SPM12 (Wellcome Trust Center for Neuroimaging) on MATLAB 2013b to process MRI images of all PDD, PDND, and HC participants. VBM analysis encompassed image segmentation and spatial normalization to obtain relative volume maps of gray matter, white matter, and cerebrospinal fluid in the standard space using diffeomorphic anatomical registration through exponentiated lie algebra (DARTEL) algorithm.^17^ SBM analysis included image segmentation, cortical reconstruction, topology correction, spherical registration, and spatial normalization using the DARTEL algorithm, resulting in the central surface of the left and right brain hemispheres separately.^18^ (3) Quality checks were conducted on the acquired images, involving visual inspection and SPM data quality checks. (4) The “Surface tool” function in Cat12 was employed to extract GI, FD, and sulcal depth from the central surfaces of the left and right brain hemispheres. (5) Smoothed of the images was performed using an 8 mm Gaussian kernel for GMV and a 20 mm Gaussian kernel for GI, FD, and sulcal depth. All procedures were carried out following the guidelines provided in the Cat12 software manual.

### 2.4 Statistical analysis

Statistical demographic and clinical data analysis was performed using IBM SPSS Statistics 25.0 (SPSS Inc.). The Shapiro–Wilk test for normality was performed on all data. Age and years of education were compared among the three groups using the one‐way analysis of variance (ANOVA), while MMSE scores and HAMD‐17 scores were assessed using the Kruskal–Wallis *H* test. The age of onset between the PDD and PDND groups was compared using the independent‐samples *t*‐test, while disease duration, UPDRS‐3 scores, and modified H‐Y stage were analyzed using the Mann–Whitney *U* test. The gender ratio among the three groups was analyzed using the Chi‐square test. The statistical significance threshold was set at *p* < 0.05. Normally distributed data were reported as mean ± standard deviation (SD), while non‐normally distributed data were presented as median (lower quartile and upper quartile).

Statistical analysis of smoothed GMV and cortical complexity indices were performed using Cat12/SPM12 and SPSS 25.0. First, ANOVA was conducted on the GMV and cortical complexity indices using Cat12/SPM12, and the familywise error rate (FWE) with a statistical threshold of *p* < 0.05 was used for multiple comparison corrections. Age, sex, and education level were selected as regular covariates, and total intracranial volume (TIV) was additionally included as a covariate when performing ANONA analysis of GMV according to the Cat12 software manual. Next, the raw data from significant clusters were extracted, and post hoc comparisons were performed using Bonferroni correction in SPSS 25.0 (*p* < 0.05/3 = 0.017). Finally, Spearman's correlation coefficient was used to evaluate the correlations between HAMD‐17 scores and morphological changes (GMV, GI, FD, and sulcal depth) in PDD group using a two‐tailed comparison and a significance level of *p* < 0.05.

## 3 RESULTS

### 3.1 Descriptive analysis

The demographic and clinical data of all participants are shown in Table 1. There were no significant differences observed among the PDD, PDND, and HC groups regarding gender, age, education level, and MMSE scores. The PDD and PDND groups were well matched in terms of disease duration, age at onset, UPDRS‐III scores, and modified H‐Y stage. Moreover, the HAMD‐17 scores in the PDD group were significantly higher than those in the other two groups (*p* < 0.001).

**TABLE 1:** The demographic and clinical data of all participants.

	PDD (*N* = 36)	PDND (*N* = 41)	HC (*N* = 38)	*p* value
Sex (male/female)	16/20	25/16	19/21	0.295^a^
Age (years)	59.69 ± 7.40	58.20 ± 10.37	56.95 ± 7.18	0.375^b^
Education (years)	6.76 ± 4.56	8.18 ± 4.39	8.11 ± 3.42	0.251^b^
Disease duration (years)	2.00 (1.00, 3.88)	2.00 (1.00, 3.00)	NA	0.339^c^
Age of onset (years)	56.70 ± 7.11	55.36 ± 10.17	NA	0.511^d^
MMSE	26.00 (24.00, 29.00)	27.00 (25.00, 29.00)	28.00 (24.00, 39.75)	0.408^e^
UPDRS‐III	24.00 (16.00, 33.25)	18.00 (9.00, 30.00)	NA	0.145^c^
Modified H‐Y	2.00 (1.63, 2.50)	2.00 (1.25, 2.50)	NA	0.155^c^
HAMD‐17	13.50 (11.00, 19.00)	2.00 (1.00, 4.00)	1.00 (0, 3.00)	<0.001^e^

*Note*: Data are presented as mean ± standard deviation, median (lower quartile and upper quartile) for continuous variables, or frequencies for categorical ones. For comparisons of demographics: ^a^ *p* value for the gender difference was obtained by chi‐square test. ^b^ *p* values were obtained by one‐way analysis of variance (ANOVA) tests. ^c^ *p* value was obtained by Mann–Whitney *U* test. ^d^ *p* values were obtained by two‐sample *t*‐test. ^e^ *p* values were obtained by Kruskal–Wallis *H* test.

Abbreviations: H&Y, Hoehn & Yahr staging scale; HAMD‐17, 17‐item Hamilton Depression Rating Scale; HC, healthy control; MMSE, Mini‐Mental State; NA, not applicable; PDD, Parkinson's disease with depression; PDND, PD patients without depression; UPDRS‐III, Unified Parkinson's Disease Rating Scale–motor part III.

### 3.2 Gray matter volume analysis

**FIGURE 1:**
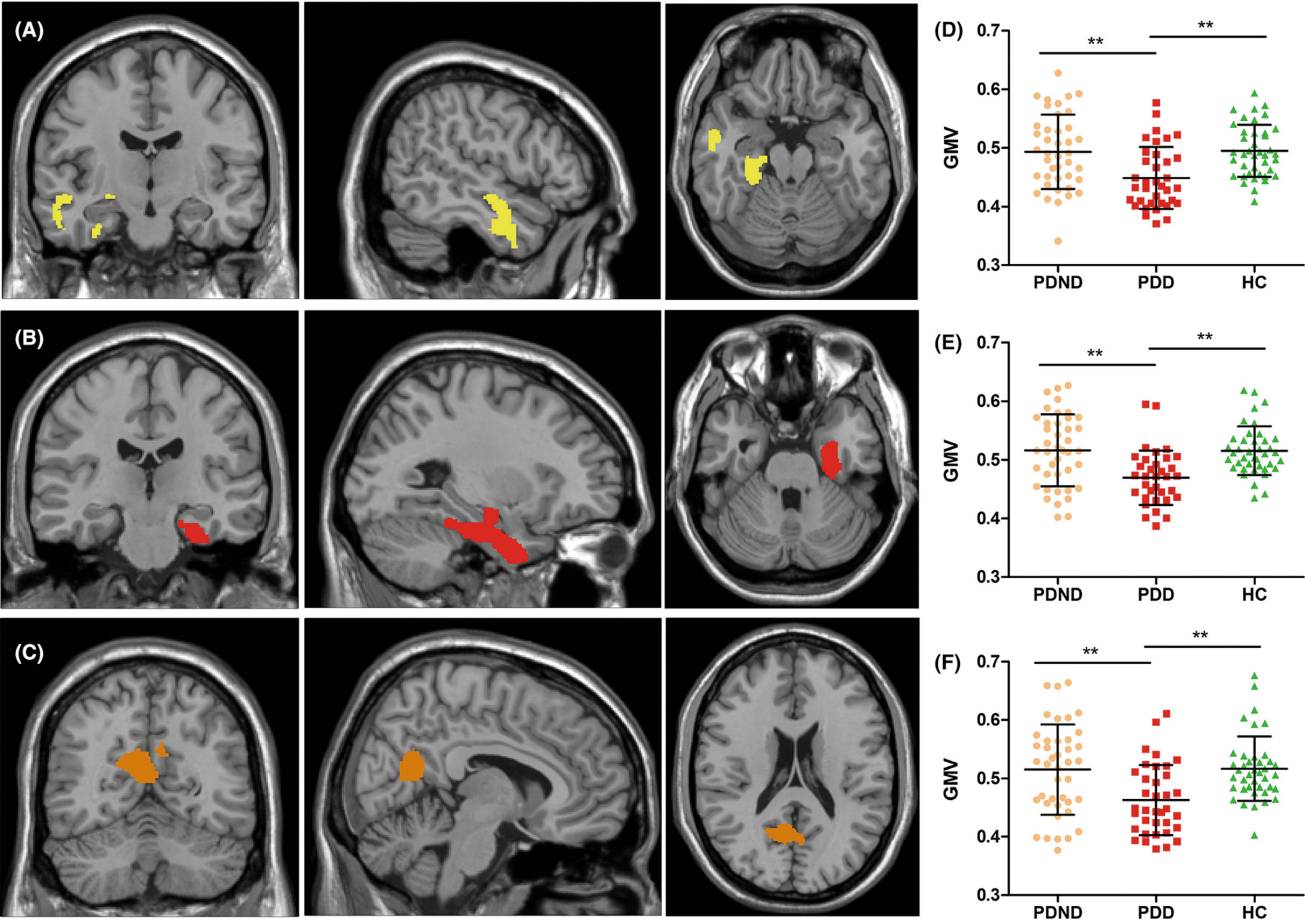
(A–C) Gray matter volume (GMV) reduction regions in Parkinson's disease with depression (PDD) (*p* < 0.05, family‐wise error correction). (A) Figure of the cluster with the peak point located in the right middle temporal lobe. (B) Figure of the cluster with the peak point located in the left parahippocampal gyrus. (C) Figure of the cluster with the peak point located in the left precuneus. (D) Plot of the GMV distribution in the cluster with the peak point located in the right middle temporal lobe. Post hoc analysis revealed a significantly decreased GMV in the PDD group compared with the PD patients without depression (PDND) group (**p = 0.001) and the healthy control (HC) group (**p = 0.001). (E) Plot of the GMV distribution in the cluster with the peak point located in the left parahippocampal gyrus. Post hoc analysis revealed a significantly decreased GMV in the PDD group compared with the PDND group (**p < 0.001) and the healthy controls (HC) group (**p < 0.001). (F) Plot of the GMV distribution in the cluster with the peak point located in the left precuneus. Post hoc analysis revealed a significantly decreased GMV in the PDD group compared with the PDND group (**p = 0.002) and the HC group (**p = 0.001).

In comparison with HC and PDND, PDD exhibited widespread GMV reductions. Specifically, significant GMV reductions were observed in the bilateral medial parietal, occipital and temporal cortex (precuneus, lingual, calcarine, and bilateral fusiform cortex), right dorsolateral temporal cortex (middle and inferior temporal cortex), bilateral parahippocampal cortex, and bilateral hippocampus cortex. No significant GMV differences were found between PDND and HC groups. Please refer to Table 2 and Figure 1 for a visual representation of the GMV reduction observed in PDD patients.

**TABLE 2:** VBM Results: Regions of gray matter volume (GMV) reduction in Parkinson's disease with depression (PDD) compared to healthy controls (HC) and Parkinson's disease without depression (PDND) (*p* < 0.05, family‐wise error correction).

Anatomic region	Cluster sizes	*F*‐Value	MNI coordinates		
			*X*	*Y*	*Z*
Temporal_Mid_R	2777	17.63	54	−9	−23
*Fusiform_R*		13.90	29	−29	−21
*Temporal_Pole_Mid_R*		11.45	24	5	−38
*Temporal_Inf_R*		10.85	41	6	−36
*ParaHippocampal_R*		10.71	33	−32	−11
*Hippocampus_R*		10.45	35	−35	−6
Parahippocampa_L	2360	14.31	−27	−18	−27
*Fusiform_L*		11.76	−26	3	−39
*Hippocampus_L*		10.16	−29	−12	−17
Precuneus_L	1871	14.94	−9	−60	29
*Calcarine_R*		14.51	5	−60	15
*Lingual_L*		3.81	−5	−77	0

*Note*: Secondary peaks are in italics.

Abbreviations: HC, healthy control; L, left; PDD, Parkinson's disease with depression; PDND, PD patients without depression; R, right.

### 3.3 Cortical complexity analysis

**FIGURE 2:**
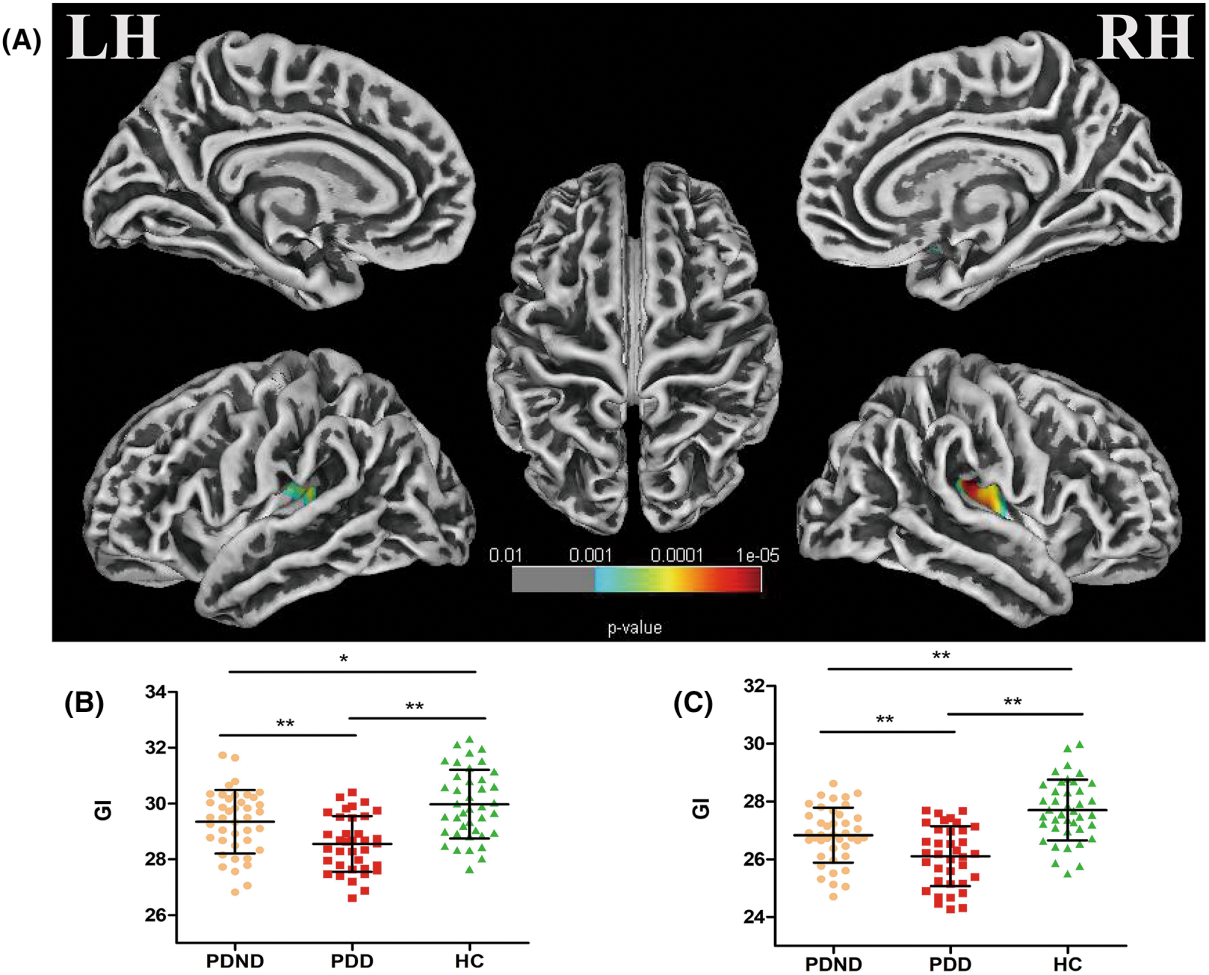
(A) Gyrification index (GI) reduction regions in Parkinson's disease with depression (PDD) (*p* < 0.05, family‐wise error correction). (B) Plot of the GI distribution in the cluster in the left hemisphere. Post hoc analysis revealed a significantly decreased GI in the PDD group compared with the PD patients without depression (PDND) group (**p = 0.008) and the healthy control (HC) group (**p < 0.001). Additionally, GI values decreased in the PDND group compared to HC (*p = 0.04). (C) Plot of the GI distribution in the cluster in the right hemisphere. Post hoc analysis revealed a significantly decreased GI in the PDD group compared with the PDND group (**p = 0.006) and the HC group (**p < 0.001). Additionally, GI values decreased in the PDND group compared to HC (**p = 0.01).

GI analysis revealed a significant cluster in the left hemisphere, comprising 920 vertices (*F* = 10.45, *p* = 0.00007, FWE corrected). This cluster is located in the insula, transverse temporal gyrus, and superior temporal gyrus. Post hoc analysis demonstrated a significantly decreased GI in the PDD group compared to both the PDND group (*p* = 0.008, Bonferroni corrected) and the HC group (*p* < 0.001, Bonferroni corrected). Additionally, a significantly decreased GI cluster was observed in the right hemisphere, comprising 3206 vertices (*F* = 13.56, *p* = 0.00001, FWE corrected) with a major mainly presence in the insula, superior temporal gyrus, and supramarginal gyrus. Post hoc analysis indicated a significantly decreased GI in the PDD group compared to both the PDND group (*p* = 0.006, Bonferroni corrected) and the HC group (*p* < 0.001, Bonferroni corrected). Further details on the clusters can be found in Table 3 and Figure 2. Notably, there were no statistically significant differences observed in sulcal depth and FD among the PDD, PDND, and HC groups.

**TABLE 3:** SBM Results: Regions of decreased gyrification index (GI) in Parkinson's disease with depression (PDD) compared to healthy controls (HC) and Parkinson's disease without depression (PDND) (*p* < 0.05, family‐wise error correction).

Hemisphere	Overlap of atlas region	Cluster size	*p*‐value	*F*‐value	Peak MNI coordinates		
					*X*	*Y*	*Z*
LH	73% insula	920	0.00007	10.45	−37	−17	15
	23% transverse temporal						
	4% superior temporal						
RH	63% insula	3206	0.00001	13.56	45	6	−18
	18% superior temporal						
	8% supramarginal						
	7% postcentral						
	3% transverse temporal						

*Note*: Atlas labeling was performed according to the Desikan–Killiany atlas.

Abbreviations: LH, left hemisphere; RH, right hemisphere.

### 3.4 Correlational analysis

The correlation between the GMV of left precuneus and HAMD‐17 scores in the PDD group tended to be significant (*r* = −0.318, *p* = 0.059). The GMV of the right middle temporal gyrus (*r* = −0.259, *p* = 0.128), and the GMV of the left parahippocampal gyrus (*r* = −0.094, *p* = 0.586) did not significantly correlate with scale scores. The GI of the left cluster (*r* = −0.187, *p* = 0.275) and the GI of the right cluster (*r* = −0.106, *p* = 0.537) also did not significantly correlate with scale scores.

## 4 DISCUSSION

Our research systematically investigated changes in GMV and cortical complexity in PDD patients, utilizing SBM and VBM methods. Our investigation yielded the following key findings: (1) Extensively GMV loss was observed in PDD patients primarily in the bilateral medial parietal–occipital–temporal cortex (precuneus, lingual, calcarine, and fusiform cortex), right dorsolateral temporal cortex (middle and inferior temporal cortex), bilateral parahippocampal cortex, and bilateral hippocampus cortex. (2) PDD patients exhibited decreased GI in the bilateral insula and dorsolateral temporal cortex when compared to PDND and HC. These findings may contribute to our understanding of the potential mechanisms of depression in PD from a morphological perspective.

### 4.1 The abnormal cortical structure in the temporal–occipital lobe is related to depression in PD

Both VBM and SBM analyses consistently confirmed cortical abnormalities in the temporal–occipital lobe in PDD. These abnormalities include atrophy of the medial temporal–occipital cortex and decreased GI in the superior and middle temporal gyri. This is consistent with the results of previous studies on GMV.^7^, ^8^ In a longitudinal follow‐up study conducted by Hanganu et al.,^12^ they identified a negative correlation between cortical thickness in the right temporo‐occipital junction, right medial occipital lobe, and left middle temporal gyrus, and the scores on the Beck Depression Inventory‐II scale (BDI‐II) in 24 non‐demented PD patients. Moreover, they also observed that PD patients with higher baseline BDI scores had a faster reduction in cortical thickness in the left middle temporal gyrus.^12^ Yin et al.^10^ also found extensive cortical atrophy, affecting the frontal, occipital, and temporal cortex, as well as alterations in functional connectivity within these atrophic regions in PDD patients. An investigation of functional brain networks revealed disruptions and reorganization of the temporal–occipital visual cortex network in PDD patients, compared to HC and PDND.^25^ Previous research strongly indicated the involvement of structural and functional changes in the temporal–occipital cortex in PD‐related depression. As we know, the primary pathological characteristic of PD is the accumulation of Lewy bodies, which are rich in α‐synuclein, and the loss of dopaminergic neurons. According to Braak's hypothesis, during stages 5 and 6 of PD progression, Lewy body deposition extends beyond the classical involvement in the substantia nigra and affects the limbic system and the cerebral neocortex,^26^ resulting in structural and functional abnormalities in these regions. Our findings provided further evidence of the structural abnormalities in the temporal–occipital neocortex in PDD. The temporal–occipital cortex is primarily responsible for the transmission and processing of visual information in healthy individuals.^27^ Therefore, we speculate that the extensive deposition of Lewy bodies, which damage cortical neurons, leads to structural and functional impairments in the temporal–occipital cortex, consequently affecting the visual processing of emotional information. This may render patients more susceptible to negative emotions such as fear and sadness. Additionally, the chronic burden of the disease itself, coupled with physical and mental exhaustion, makes PD patients more prone to experiencing negative emotions that can lead to depression.

### 4.2 Abnormalities in perihippocampal structures are involved in the development of PD‐related depression

Our research findings also demonstrated a decrease in GMV in the bilateral hippocampus and parahippocampal gyrus, which has been confirmed by previous studies.^6^, ^7^, ^8^ The hippocampus, because of its involvement in learning and memory processes, has long been associated with cognitive impairment in PD patients.^28^ However, evolving research has established that hippocampal atrophy is not only linked to the development of dementia but also to PD‐related depression.^29^, ^30^ Studies on MDD have shown that chronic stress leads to excessive activation of the hypothalamic–pituitary–adrenal (HPA) axis in the human body.^31^ Prolonged exposure to corticosteroids can decrease neurogenesis in the hippocampal region, leading to hippocampal atrophy.^30^ Similarly, we also found excessive HPA axis activation in PDD.^31^ This may be the reason for hippocampal atrophy in PDD. Additionally, Ricci et al.^29^ found that PDD exhibited reduced levels of serum brain‐derived neurotrophic factor (BDNF), which were lower than those of both depression patients and PD patients, and antidepressant treatment could help improve this situation by restoring their BDNF levels to normal. BDNF is widely distributed in the central nervous system, especially in the cerebral cortex and hippocampus. It can promote neurogenesis in the hippocampus as well as maintain the activity of dopamine neurons. Thus, the reduction in BDNF may also contribute to hippocampal atrophy in PD patients. Hence, the pathological and physiological mechanisms underlying depression in PD patients are complex. In addition to Lewy body deposition in PD patients, chronic stress and neurotrophic factors may also play a role because of their impact on the hippocampus.

### 4.3 Abnormalities in bilateral medial parieto‐occipital cortical structures, especially precuneus, involved in the development of PDD

Our study found a reduction in GMV in the bilateral precuneal gyrus, and the correlation between GMV and HAMD‐17 scores tended to be significant (*p* = 0.059). The precuneal gyrus is the medial part of the parietal lobe and is a crucial part of the default mode network (DMN). The DMN exhibits robust spontaneous neural activity during the resting state but is inhibited when engaging in attention‐demanding tasks. This network is responsible for both intrinsic and extrinsic attention allocation and self‐reference, and it is negatively correlated with the task‐positive network which is primarily involved in processing external stimuli.^32^ It is widely recognized that the DMN plays a significant role in depression. In individuals with depression, this network is often found to be over‐activated or over‐connected, making it difficult to move attention from the internal world to external stimuli and preventing patients from getting rid of their thoughts and emotions.^32^ A previous study investigating cortical structures in PDD has mentioned the precuneal gyrus, noting the increase in cortical thickness among PDD patients.^33^ This result differs from our findings, possibly due to variations in the duration of depression. It is generally understood that in the early stages of depression, compensatory inflammatory responses can lead to increased cortical thickness in the precuneal gyrus, while prolonged depressive states may result in cortical atrophy. Our previous study on brain functionality supports our current discoveries, indicating increased compensatory functional connectivity within the DMN among PDD patients, with notable differences mainly in the right precuneal gyrus.^34^ Despite the contradictory findings in existing research regarding the precuneal gyrus, it is generally acknowledged that this region plays a significant role in the pathophysiology of PD‐related depression, which is closely associated with abnormalities in the DMN network responsible for intrinsic emotion processing in the brain.

### 4.4 Altered GI in the insular cortex is associated with depressive symptoms in PD

Our SBM analysis revealed significantly decreased GI in the bilateral insular cortex of PDD patients when compared to both HC and PDND patients. The insular cortex plays a pivotal role as an anatomical integration center, receiving sensory information input from both internal and external sources and establishing extensive connections with various cortical and subcortical brain regions.^35^ This collective integration contributes to sensory, emotional, motivational, and cognitive functions.^35^ Given its status as a crucial emotional hub in the brain, damage to the insular cortex directly leads to abnormalities in emotional regulation. The GI, a measure of cortical complexity, quantifies the amount of cortex buried within the sulcal folds as compared with the amount of cortex on the outer visible cortex.^36^ High GI indicates extensive folding of the cortex, while low GI indicates limited folding. According to the tension‐based theory of cortical morphogenesis, decreased GI implies a reduction in deep white matter fibers, as tension along the axons in white matter is the primary driving force for cortical folding.^20^ Diffusion tensor imaging studies have shown impaired white matter fiber integrity and connectivity in the uncinate fasciculus and inferior fronto‐occipital fasciculus, which are located in and around the deep insula, in PDD.^37^, ^38^ Hence, we speculate that the reduced GI in the insula region reflects damage to deep white matter fibers, likely leading to abnormal emotional information transmission and depressive mood among PDD patients.

Our study found no significant differences in GMV between PDND and HC, in contrast to previous research. Previous studies reported GMV reductions in various cerebral cortex regions of PD patients compared to HC.^39^ Differences in grouping criteria and sample heterogeneity may impact the generalizability of our findings. The regions with GMV differences identified in previous studies were mainly related to cognitive, motor functions and depression in PD patients. To focus on depression, we matched three groups in our study for education level and MMSE scores, eliminating the interference of cognitive function. Additionally, since patients with severe depressive symptoms often exhibit severe motor symptoms, the PDND group included in the study was in the early stages of motor dysfunction, with a modified H‐Y stage not exceeding 2.5 and UPDRS‐III scores not exceeding 30. Therefore, the PDND group consisted of individuals in the early stages of cognitive and motor impairments. This could explain why our study did not find differences in GMV between the PDND and HC. Instead, we observed significant decreases in GI in the bilateral insular regions of the PDND group compared to HC, indicating that structural brain changes occur before clinical depressive symptoms. It also indicates that GI is more sensitive to cortical structural changes compared to GMV, further emphasizing the importance of studying cortical complexity indices.

### 4.5 Limitations

Several limitations to our study should be acknowledged. First, all study participants were recruited from the Neurology Department and sought medical attention primarily for PD‐related motor symptoms. This implies that it is challenging for us to obtain brain MRI images from PD patients in the early stages of the illness when depression is the initial symptom and motor symptoms have not yet manifested. To address this limitation, it would be beneficial to conduct long‐term longitudinal follow‐up studies with collaboration among the radiology, psychology, and psychiatry departments. Furthermore, our study participants discontinued anti‐PD medications for 12 hours before undergoing MRI scans. However, this may not entirely eliminate the influence of medication, since it is known that anti‐PD medications can improve depressive symptoms in patients.^40^ Finally, in the PDD group, the correlation between the GMV of left precuneus and HAMD‐17 scores tends to be significant. Subsequent research may require a larger sample size to confirm the correlation.

## 5 CONCLUSION

Our research findings indicated that PDD patients exhibited more extensive and pronounced cortical atrophy compared to PDND patients, primarily concentrated in the parietal–occipital–temporal lobes, parahippocampal gyrus, and hippocampus. We also observed a decrease in GI in the insular and dorsolateral temporal cortex. These findings provide morphological support for our understanding of the pathophysiological mechanisms underlying depression in PD.

## CONFLICT OF INTEREST STATEMENT

The authors declare that the research was conducted in the absence of any commercial or financial relationships that could be construed as a potential conflict of interest.

## Supporting information


Data S1.

## Data Availability

The data that support the findings of this study are available from the corresponding author upon reasonable request.
