# Supplementary material for: Alterations in cortical volume and complexity in Parkinson's disease with depression
Source: CNS Neurosci Ther. 2024 Feb 8;30(2):e14582. doi: 10.1111/cns.14582 (PMC10851315; doi:10.1111/cns.14582)

1. Full unedited images for Figure 1

a. Figure of the cluster with the peak point located in the right middle temporal lobe.

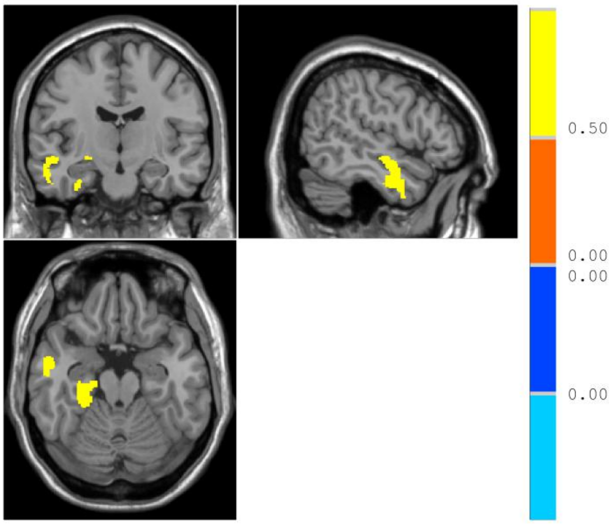

b. Figure of the cluster with the peak point located in the left parahippocampal gyrus.

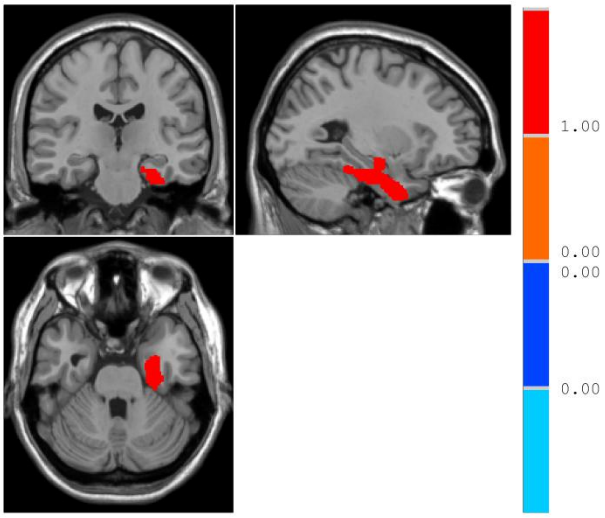

c. Figure of the cluster with the peak point located in the left precuneus.

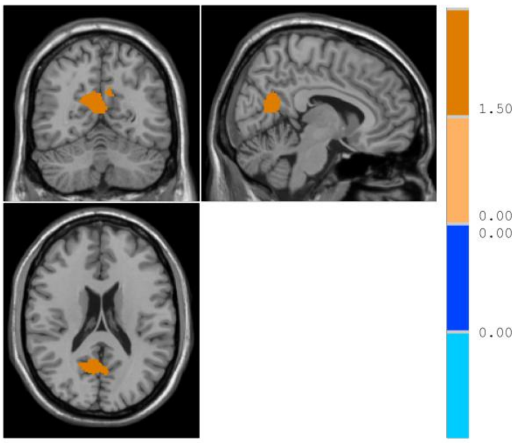

d. Plot of the GMV distribution in the cluster with the peak point located in the right middle temporal lobe.

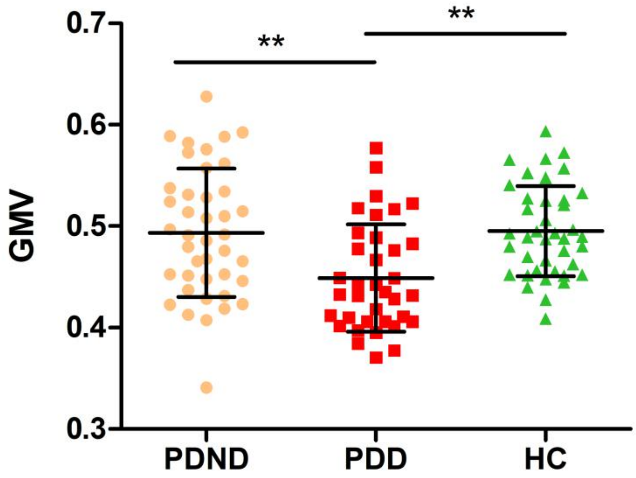

e. Plot of the GMV distribution in the cluster with the peak point located in the left parahippocampal gyrus.

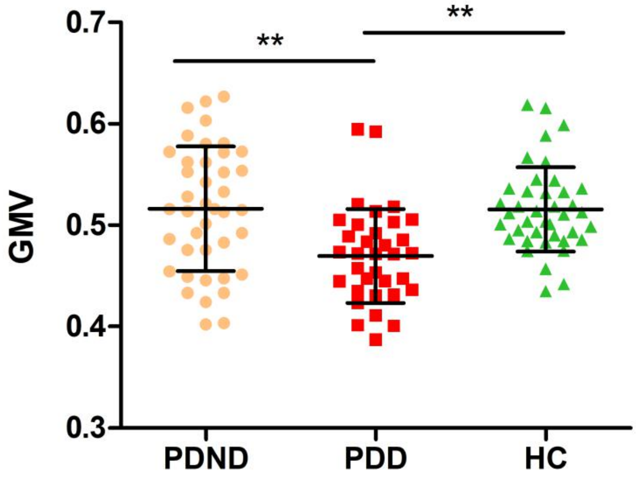

f. Figure of the GMV distribution in the cluster with the peak point located in the left precuneus.

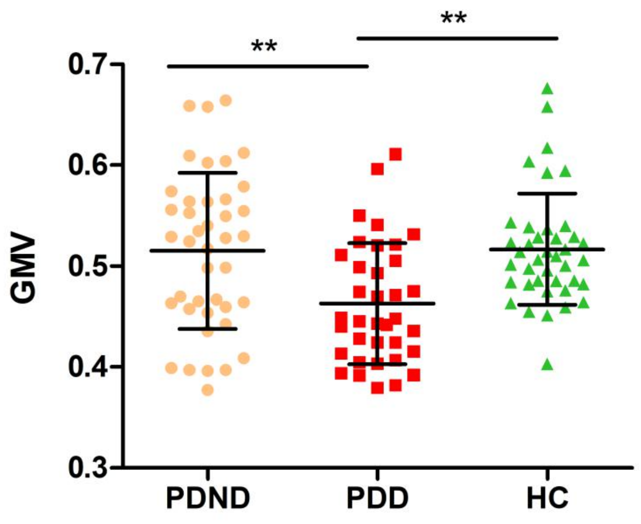

2. Full unedited images for Figure 2

a. Gyrification index (GI) reduction regions in Parkinson's disease with depression (PDD) .

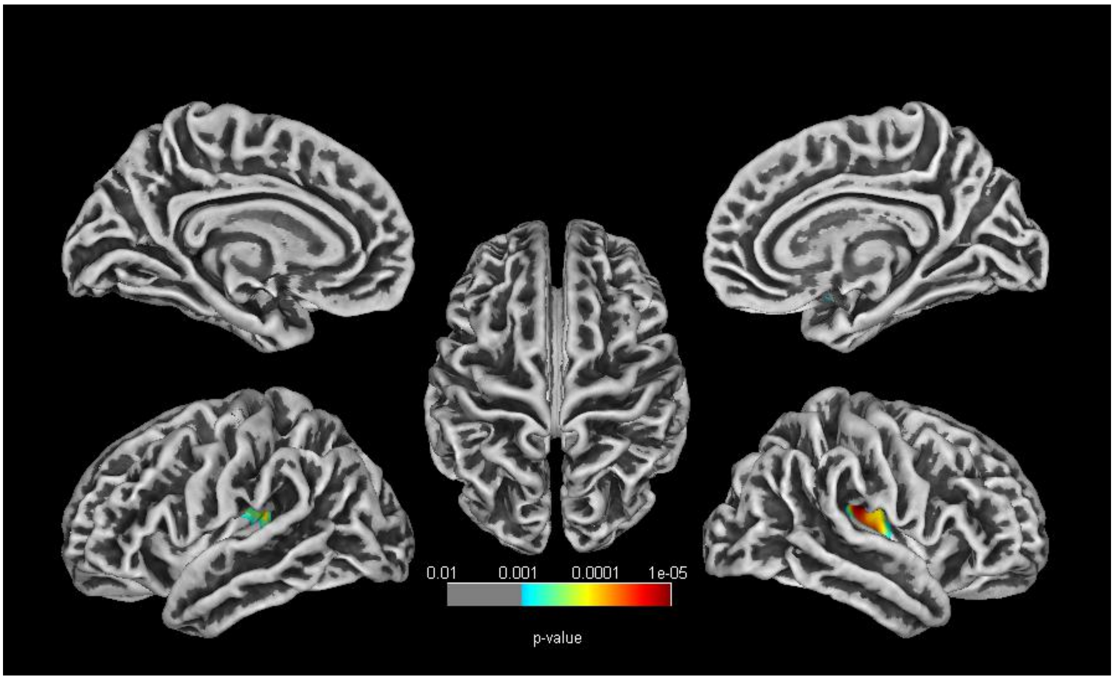

b. Plot of the GI distribution in the cluster in the left hemisphere.

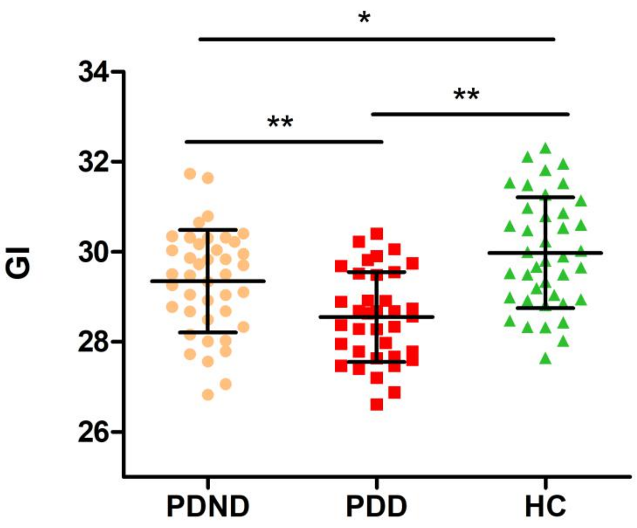

c. Plot of the GI distribution in the cluster in the right hemisphere.

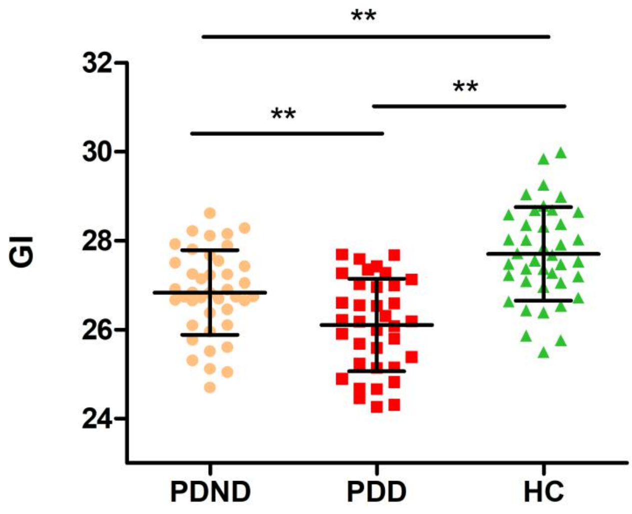

Supplement: Supplementary file 1 — Data S1. [file CNS-30-e14582-s001.pdf]
